# Depression, professional self-efficacy, and job performance as predictors of life satisfaction: the mediating role of work engagement in nurses

**DOI:** 10.3389/fpubh.2024.1268336

**Published:** 2024-02-01

**Authors:** Wilter C. Morales-García, María Vallejos, Liset Z. Sairitupa-Sanchez, Sandra B. Morales-García, Oriana Rivera-Lozada, Mardel Morales-García

**Affiliations:** ^1^Unidad de Ciencias Empresariales, Escuela de Posgrado, Universidad Peruana Unión, Lima, Peru; ^2^Escuela Profesional de Medicina Humana, Facultad de Ciencias de la Salud, Universidad Peruana Unión, Lima, Peru; ^3^Facultad de Teología, Universidad Peruana Unión, Lima, Peru; ^4^Sociedad Científica de Investigadores Adventistas (SOCIA), Universidad Peruana Unión, Lima, Peru; ^5^Business Sciences Unit, Graduate School, Universidad Peruana Unión, Lima, Peru; ^6^Universidad Peruana Unión, Tarapoto, Peru; ^7^Escuela Profesional de Psicología, Facultad de Ciencias de la Salud, Universidad Peruana Unión, Lima, Peru; ^8^Departamento Académico de Enfermería, Obstetricia y Farmacia, Facultad de farmacia y Bioquímica, Universidad Científica del Sur, Lima, Peru; ^9^South American Center for Education and Research in Public Health, Universidad Norbert Wiener, Lima, Peru; ^10^Unidad de Posgrado de Ciencias de la Salud, Escuela de Posgrado, Universidad Peruana Unión, Lima, Peru

**Keywords:** work engagement, depression, self-efficacy, job performance, life satisfaction

## Abstract

**Background:**

The life satisfaction and job performance of nursing professionals are affected by a multitude of factors, including work engagement, self-efficacy, and depression. The Job Demands-Resources (JD-R) model provides a theoretical framework to explore these relationships.

**Objective:**

Our study aimed to analyze the primary goal of this research, which is to examine the mediating role of work engagement in the relationship between depression, professional self-efficacy, job performance, and their impact on life satisfaction in nurses, using the JD-R theory as a guide.

**Methods:**

This cross-sectional study involved 579 participants aged between 21 to 57 years (*M* = 39, SD = 9.95). Mediation analysis was used to examine the influence of depression, self-efficacy, and job performance on work engagement, and in turn, its effect on life satisfaction.

**Results:**

Findings indicated that work engagement plays a crucial mediating role between depression, self-efficacy, job performance, and life satisfaction. Interventions to increase work engagement could assist nurses in better managing depression and improving their performance and life satisfaction.

**Conclusions:**

Our study highlights the need for workplace policies and strategies that foster work engagement and self-efficacy among nurses while effectively managing job demands to prevent depression. Moreover, these findings underscore the importance of the JD-R theory to understand and improve nurses' job satisfaction and performance, and suggest areas for future research, including exploring other potential factors and applying these findings across different contexts and cultures.

## 1 Introduction

The work environment and life satisfaction have an essential relationship in wellbeing and a determining influence on mental health and quality of life. The relevance of this connection is particularly evident in daily interactions and people's functionality in their work activities (1, 2). In this context, factors such as depression, professional self-efficacy, and job performance become especially relevant as they can either boost or hinder workers' life satisfaction (3). This reality manifests with particular intensity in the nursing profession in Peru, a vital pillar of the nation's health system (4, 5). However, Peruvian nurses face a host of challenges and job demands that result in a significant impact on their life satisfaction. These obstacles include staff shortages, resource insufficiency, work stress, high turnover rates, and heavy workloads. Each of these factors, both individually and collectively, has the potential to negatively influence their psychological wellbeing, and consequently, their ability to provide quality patient care (6–8).

The Job Demands-Resources (JD-R) theory provides a robust conceptual framework that allows the understanding of the dynamics between job demands and resources and their eventual influence on crucial aspects such as work engagement and life satisfaction. Job demands, such as tension stemming from depression situations, emerge as strain factors that can exhaustively affect workers' personal resources, pushing them toward tension and health deterioration (9, 10). On the other hand, job resources, exemplified in job performance, as well as personal resources, notably work self-efficacy, function as driving forces that enable the achievement of objectives, promoting personal and professional growth. They become catalysts for motivation and productivity, aspects that essentially transcend the work environment and influence individuals' personal spheres (10–12). In the JD-R theory, work engagement holds particular relevance. This component plays a fundamental mediating role, positioning itself at the core of the relationship between job demands and resources and job outcomes (13, 14). In the specific case of Peruvian nurses, understanding the mediating role of work engagement becomes essential as this understanding illuminates the interconnections between individual and work factors and their correlation with their life satisfaction.

At this point, it is necessary to emphasize that the JD-R theory not only recognizes the potential effects of work factors but also takes into account the relevance of personal resources, such as work self-efficacy, in the job dynamics (10, 15–17). These resources can trigger an active coping process against job demands and improve the utilization of available work resources. Therefore, the continuous dialogue between job demands and resources, both job-related and personal, is a cross-cutting axis that shapes individuals' work experience. Hence, in the context of Peruvian nurses, understanding the mediating role of work engagement will provide valuable information about how individual and work factors relate to their life satisfaction.

Despite the growing attention paid to the relationship between these factors, less attention has been paid to work engagement in these relationships and how these variables can jointly affect life satisfaction in a specific work context, such as that of nurses. The current picture concerning this issue is certainly worrisome, as global and national data have shown a high prevalence of depression and a low level of life satisfaction among nursing professionals (18, 19). These conditions can result in negative effects both at the individual and organizational levels, including emotional exhaustion, job performance deterioration, and decreased quality of care provided to patients (20).

While growing awareness about the importance of mental health and wellbeing in the workplace has motivated the implementation of various interventions to address these issues (21), there still exists a lack of studies specifically addressing the mediating role of work engagement in these relationships and how these factors interact in specific work contexts.

Therefore, this research seeks to fill this gap in the literature and contribute to a deeper understanding of these factors. This will help identify effective interventions that can improve nurses' emotional wellbeing and life satisfaction (22, 23). In this sense, the research will contribute to a deeper understanding of these factors and help identify effective interventions that can improve nurses' emotional wellbeing and life satisfaction. Therefore, the main objective of this research is to analyze the mediation of work engagement in the relationship between depression, professional self-efficacy, job performance, and their impact on life satisfaction in nurses.

### 1.1 Literature review

#### 1.1.1 Life satisfaction

Life satisfaction is a broad construct that refers to a person's overall evaluation of their life in general and their subjective well-being (24). It is associated with elements such as work engagement, performance, and self-efficacy (25, 26), as well as with job and personal demands and resources (3, 27). In this context, job resources, including self-efficacy and performance, positively impact life satisfaction, while demands, such as depression, have adverse effects (15, 28, 29). This dynamic is particularly relevant in high-stress environments, like the healthcare sector, where work-life imbalance and emotional exhaustion are common (30–32). Studies show a correlation between high work engagement and increased life satisfaction, as well as a negative association with burnout and depressive symptoms. Moreover, it was observed that job resources have positive effects on work engagement and life satisfaction, and negative effects on burnout and depressive symptoms. On the other hand, a high workload is associated with more burnout and depressive symptoms, and less life satisfaction (3). There exists an interplay between work engagement and burnout, and between life satisfaction and depressive symptoms, all influencing occupational health outcomes like recovery, work addiction, and mental health diagnoses (33). Finally, the JD-R model indicates that burnout and work engagement are interrelated and contribute to other occupational health outcomes, reflecting in the overall well-being of employees (34). This approach highlights the variability of the impacts of demands and resources throughout life, suggesting that optimizing personal and professional development may be key to improving life satisfaction and reducing depressive symptoms at different stages of a professional's career.

#### 1.1.2 Depression

Depression is a common mental disorder characterized by symptoms like persistent sadness and loss of interest (35). It is considered a job demand that negatively impacts performance and job satisfaction (36, 37). Burnout, associated with job strain and chronic emotional demands, is linked to negative work outcomes and health problems such as depression and anxiety (10, 38, 39). Job strain and burnout, particularly in human service professionals, can increase the risk of mental illnesses and physical problems, also affecting the quality of care in healthcare workers (40–44). The JD-R theory suggests that overall well-being or distress can influence how employees handle job demands and resources (15). The revised JD-R model proposes that high job demands and insufficient job resources lead to burnout and health problems like depression (10, 11). Furthermore, increased job strain can lead employees to adopt maladaptive coping strategies, exacerbating depression and anxiety (45).

#### 1.1.3 Professional self-efficacy

Professional self-efficacy refers to an individual's beliefs about their ability to carry out tasks and overcome challenges in their work environment. This confidence in one's abilities to organize and execute actions aimed at achieving work goals has become a cornerstone for understanding workers' functioning in their workplace (46). Self-efficacy is considered a significant personal resource in the Job Demands-Resources (JD-R) model, influencing how workers cope with job demands and how they utilize available job resources. Thus, a worker with a high level of self-efficacy may perceive elevated job demands as challenges rather than threats, and more effectively leverage job resources at their disposal to face these demands (12, 47). Empirical literature has linked professional self-efficacy with a number of positive work outcomes, such as job satisfaction and better adaptation to job design, suggesting that beliefs in one's abilities are a critical influencer of wellbeing and performance at work (46).

Furthermore, in an increasingly digitalized work environment, the role of self-efficacy in managing job stress and strengthening emotional wellbeing has become crucial. According to the Job Demand-Resource model, professional self-efficacy is an essential resource for successfully coping with the inherent challenges of work, and therefore, to improve emotional wellbeing (48). In the context of nurses, this importance is accentuated, as their professional self-efficacy and emotional wellbeing are under constant pressure due to the demands of their professional practice. A weakened self-efficacy can jeopardize the quality of care provided to patients, highlighting the importance of strengthening this personal resource in these professionals (49).

#### 1.1.4 Job performance

Job performance, understood as individual behavior that adds value to an organization and contributes to its objectives, has evolved in its conceptualization. Now, it not only focuses on competence in tasks, but also includes elements such as adaptability, proactivity, and organizational citizenship behaviors (50). Additionally, it is recognized that this performance can be modulated by environmental factors, such as the importance of tasks to be performed and the social support received at the workplace (51). In this line, the Job Demands and Resources (JD-R) theory provides a framework to understand how these demands and resources in the workplace can affect both job performance and employee wellbeing (51). It is interesting how, in times of crisis, job performance can be affected by the interaction between job demands and resources, a relationship that can have a direct impact on workers' motivation and wellbeing (11, 52). Specifically for nurses, their job performance is a critical factor in ensuring patient safety. This performance can be modulated by both personal and work-related factors, such as depression and professional self-efficacy. This underlines the need to investigate and promote safe behaviors that improve the quality of care (53). On the other hand, it has been shown that high job performance, along with job satisfaction and organizational commitment, can have a positive impact on nurses' performance and on the quality of care provided to patients (54).

#### 1.1.5 Work engagement

Work engagement, defined as an emotional and cognitive state in which employees feel energetic, dedicated, and absorbed in their work (55), emerges as a key element in mediating the influences of job demands and resources on employee well-being (56). Job resources are crucial for fostering robust work engagement (57, 58). This engagement not only enhances productivity and motivation but also acts as a buffer against the negative effects of job demands, such as overload and burnout (59). In the educational context, for example, teachers' self-efficacy has shown a positive correlation with their work engagement, even in the face of challenging demands like workload and problematic student behavior (60). Work engagement also exerts a significant influence on key aspects like the intention to leave the job, job adaptation, and organizational performance. Engaged employees tend to exhibit greater loyalty and both affective and normative commitment to their organization, resulting in better performance at both individual and organizational levels (61). Furthermore, work engagement can be an indicator of the frequency of job absences, reflecting higher motivation and better overall health (10).

Depression, a particularly harmful work demand, can negatively impact work engagement and, consequently, decrease life satisfaction (12). The JD-R model proposes that various motivational and energy processes, driven by work demands and resources, determine both work engagement and burnout. Quantitative work overload is associated with burnout symptoms, whereas strong work resources, such as effective leadership, self-efficacy, and resilience, foster work engagement and life satisfaction (3). Better understanding and managing nurses' work engagement could alleviate the negative impact of depression on their life satisfaction. This claim is supported by recent research highlighting the importance of work engagement in healthcare quality and the relationship between conflict management styles and nurses' work engagement (62).

Moreover, professional self-efficacy has been proven to influence job performance. This personal resource element has been studied in relation to work engagement and life satisfaction, establishing its role as a significant influencer (25, 63). In line with the JD-R theory, it is postulated that work and personal resources have a direct impact on work engagement. However, the theory goes beyond by suggesting that psychological empowerment acts as a mediator in this relationship, providing a deeper dimension to our understanding of work and personal interactions (10, 64).

In this context, professional self-efficacy, work engagement, and life satisfaction are not only seen as interrelated factors but crucial components that exert a significant effect on job performance and, by extension, on the wellbeing of healthcare professionals (12, 65). This multidimensional relationship suggests that nurses showing high levels of professional self-efficacy may experience more intense work engagement, which in turn can enhance their life satisfaction. This is a significant notion as it underscores the synergistic relationship between these factors and how, collectively, they can drive the performance and wellbeing of healthcare professionals (25).

In this sense, work engagement may play a pivotal role as a mediator in the relationship between depression, professional self-efficacy, job performance, and life satisfaction in nurses. By addressing the factors influencing work engagement, interventions and strategies could be designed to improve life satisfaction and the overall wellbeing of nurses within the framework of the JD-R theory.

**Figure 1:**
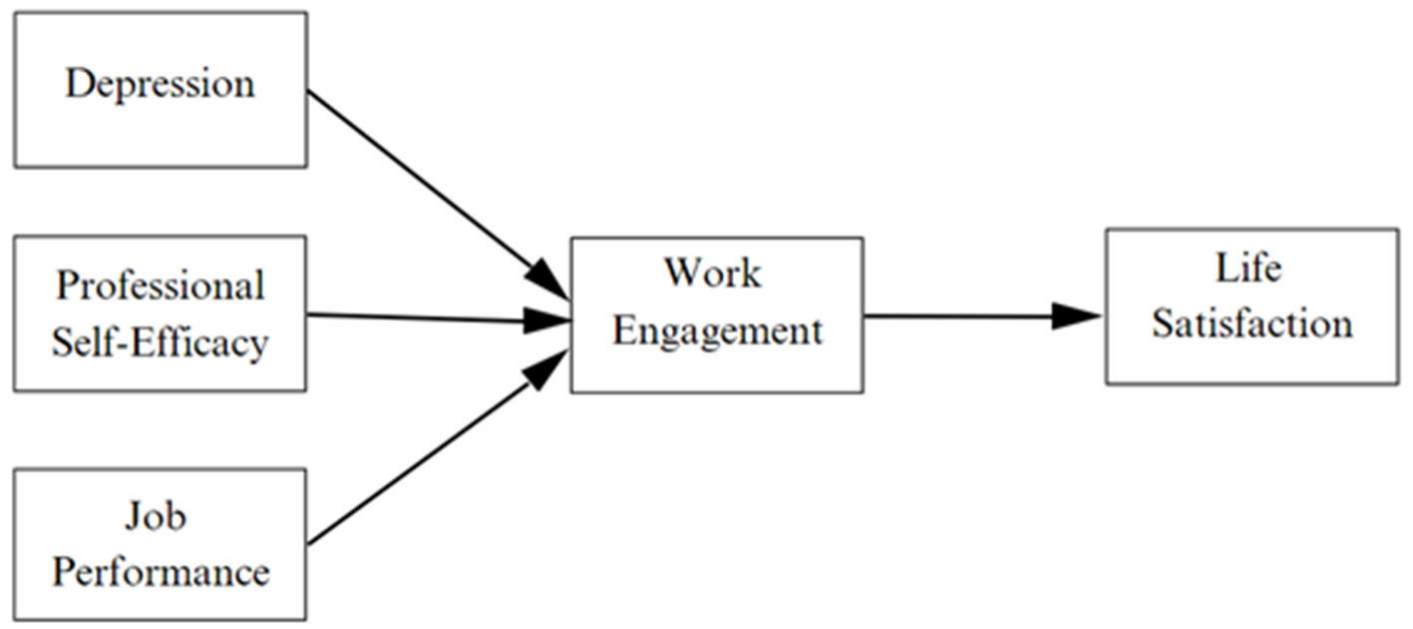
Theoretical model.

Considering the arguments presented, the following hypotheses are proposed (Figure 1):

H1: There is a negative relationship between depression and work engagement.
H2: There is a positive relationship between professional self-efficacy and work engagement.
H3: There is a positive relationship between job performance and work engagement.
H4: There is a positive relationship between work engagement and life satisfaction.
H5a: Work engagement mediates the relationship between depression and life satisfaction.
H5b: Work engagement mediates the relationship between professional self-efficacy and life satisfaction.
H5c: Work engagement mediates the relationship between job performance and life satisfaction.

## 2 Methods

### 2.1 Design and participants

The design of this study is cross-sectional and explanatory in nature, making use of a structural equation system to consider latent variables (66). In terms of sample size, the study was based on an analysis of the effect size taking into account the number of observed and latent variables in the model. Additionally, the anticipated effect size (λ = 0.3), desired statistical significance (α = 0.05), and level of statistical power (1 – β = 0.95) were considered. Based on these calculations, a minimum sample of 207 was estimated to be appropriate (67). However, for this study, we had the participation of 579 nurses from four hospitals in the Lima region, Peru, aged between 21 and 57 years (*M* = 39, SD = 9.95). For participant recruitment, a convenience sampling method was employed. The majority were female (70.5%), contracted (64.4%), and from an assisting occupational group (73.9%) (Table 1).

**Table 1:** Sociodemographic characteristics.

**Characteristics**		**Frequency**	**Percentage**
Gender	Female	408	70.5
	Male	171	29.5
Employment status	Contracted	373	64.4
	Appointed	162	28.0
	Permanent position	32	5.5
	Third-party	12	2.1
Occupational group	Administrative	151	26.1
	Caregiving	428	73.9

### 2.2 Procedure

The conduct of this study was governed by strict ethical standards and rigorous procedures. The research received approval from the Ethics Committee of a Peruvian University, under code 2023-CEUPeU-033, prior to data collection. This review and approval process ensured that our research fully complied with national and international ethical and scientific standards. Over a 3-month period, between January and March 2023, we conducted data collection. For this purpose, we invited participants to complete an online questionnaire. We used Google Forms, known for its ease of use and accessibility. Prior to data collection, it was paramount to ensure adherence to confidentiality standards and the principles of the Declaration of Helsinki, a set of ethical guidelines governing research involving human subjects. We informed each participant about the purpose of the research, and ensured that they fully understood the implications of their participation before obtaining their informed consent. This process allowed participants to make an informed decision about their involvement in the study, reinforcing our commitment to conducting ethical and respectful research.

### 2.3 Instruments

#### 2.3.1 Life satisfaction

Life satisfaction was assessed using the Spanish version of the Satisfaction with Life Scale (SWLS), a unidimensional self-report tool designed to measure life satisfaction (68). It consists of five questions, for example, “In most aspects, my life is close to my ideal”, or “I am completely satisfied with my life”, using a Likert scale ranging from 1 (strongly disagree) to 5 (strongly agree). Its reliability coefficients, omega (ω = 0.90), reflect adequate internal reliability.

#### 2.3.2 Job performance

The Spanish version of the Individual Work Performance Questionnaire (IWPQ), a self-report instrument that assesses three dimensions of job performance: task, contextual, and counterproductive behaviors was used (69). It has 13 items, for example, “I was able to do my job well because I dedicated the necessary time and effort” or “I focused on the negative aspects of the job, instead of focusing on the positive things”, and uses a Likert scale from 1 (never) to 5 (always). The Cronbach's alpha coefficients for each dimension indicate adequate internal consistency (α = 0.76 for task and contextual dimensions, and α = 0.72 for counterproductive behaviors).

#### 2.3.3 Work engagement

The Spanish version of the Brief Work Engagement Scale (UWES-9) was used (70). This scale consists of nine items, for example, “I feel full of energy in my job” or “When I wake up in the morning, I look forward to going to work”, rated on a six-point Likert scale ranging from “never” (0) to “always” (5). The scale focuses on three dimensions: vigor, dedication, and absorption. The Cronbach's alphas for these dimensions ranged from 0.84 to 0.92, indicating high internal consistency.

#### 2.3.4 Professional self-efficacy

It was measured using the Spanish version of the work self-efficacy scale, adapted from the original English version (71). This scale consists of 10 items, for example, “I will be able to find what I want in my job even if someone opposes me” or “When I find myself in a difficult work situation, I trust that I will figure out what to do”, rated on a four-point Likert scale (1 = Never, 2 = Sometimes, 3 = Often, 4 = Always). In international samples, the scale has demonstrated adequate reliability, with a Cronbach's alpha of α = 0.80 and a confirmed unidimensional structure.

#### 2.3.5 Depression

The Patient Health Questionnaire-2 (PHQ-2) depression scale was used, which consists of two items from the PHQ-9 (72). Responses are scored on a scale ranging from 0 (Not at all) to 3 (Nearly every day). This scale has shown adequate reliability, with a Cronbach's alpha of 0.72.

To measure depression, the Patient Health Questionnaire-2 (PHQ-2) scale was used. It comprises 2 elements from the PHQ-9, for example, “Feeling down, depressed, or hopeless” or “Little interest or pleasure in doing things”, with a response scale where (Not at all = 0, Several days = 1, More than half the days = 2, and Nearly every day = 3). This scale was adapted and abbreviated from the PHQ-9 and has a Cronbach's alpha of 0.72, indicating high reliability (72).

### 2.4 Statistical analysis

The statistical analysis was carried out through structural equation modeling, which allowed the evaluation of multiple relationships simultaneously. We used the MLR estimator, which is suitable for numerical variables and robust against deviations from normality (73). To assess the goodness of fit of the model, we used several statistical metrics. In particular, we employed the Comparative Fit Index (CFI), the Root Mean Square Error of Approximation (RMSEA), and the Standardized Root Mean Square Residual (SRMR). Following the guidelines set by Bentler (74), we considered CFI and TLI values >0.90 as indicative of a good model fit. For RMSEA and SRMR, values below 0.080 were interpreted as a good fit (75, 76).

The software used to perform these analyses was R (version 4.1.2), an open-source platform widely used in scientific research. Specifically, we used the “lavaan” library (version 06-10) for structural equation modeling (77).

To assess mediation, we used the “psych” package (78). Following established guidelines, we considered a variable M as a mediator between independent variable X and dependent variable Y if M is causally located between X and Y. In this case, the variable M is influenced by X, which in turn impacts Y (79, 80). The indirect effect of X on Y is thus through M (81). This allowed us to form a causal chain to compare the mediating effect of the M variables. To test the indirect effect, we applied bootstrapping with 500 iterations.

## 3 Results

### 3.1 Preliminary analysis

The results presented in Table 2 show the means (M), standard deviations (SD), and reliability coefficients (α). Cronbach's alpha (α) is above 0.7, considered as an acceptable level of internal consistency. The bivariate analysis indicated that life satisfaction shows a significant positive correlation with work engagement (*r* = 0.58, *p* < 0.01) and with professional self-efficacy (*r* = 0.62, *p* < 0.01). It is also positively correlated with job performance (*r* = 0.45, *p* < 0.01). However, life satisfaction is negatively correlated with depression (*r* = −0.55, *p* < 0.01), meaning that as life satisfaction increases, depression decreases. Similarly, work engagement is positively correlated with professional self-efficacy (*r* = 0.67, *p* < 0.01) and with job performance (*r* = 0.52, *p* < 0.01), and negatively correlated with depression (*r* = −0.50, *p* < 0.01). Also, depression shows a negative correlation with professional self-efficacy (*r* = −0.56, *p* < 0.01) and with job performance (*r* = −0.39, *p* < 0.01). Finally, professional self-efficacy shows a positive correlation with job performance (*r* = 0.58, *p* < 0.01).

**Table 2:** Descriptive statistics, internal consistencies, and correlations for the study variables.

**Variable**	***M***	**SD**	**α**	**1**	**2**	**3**	**4**	**5**
Life satisfaction	21.11	3.66	0.85	–				
Work engagement	43.36	8.27	0.88	0.58^**^	–			
Depression	0.39	1.05	0.88	−0.55^**^	−0.50^**^	–		
Professional self-efficacy	51.34	8.37	0.92	0.62^**^	0.67^**^	−0.56^**^	–	
Job performance	45.92	4.69	0.70	0.45^**^	0.52^**^	−0.39^**^	0.58^**^	–

^**^p < 0.01.

α, Cronbach's alpha.

### 3.2 Theoretical model analysis

**Figure 2:**
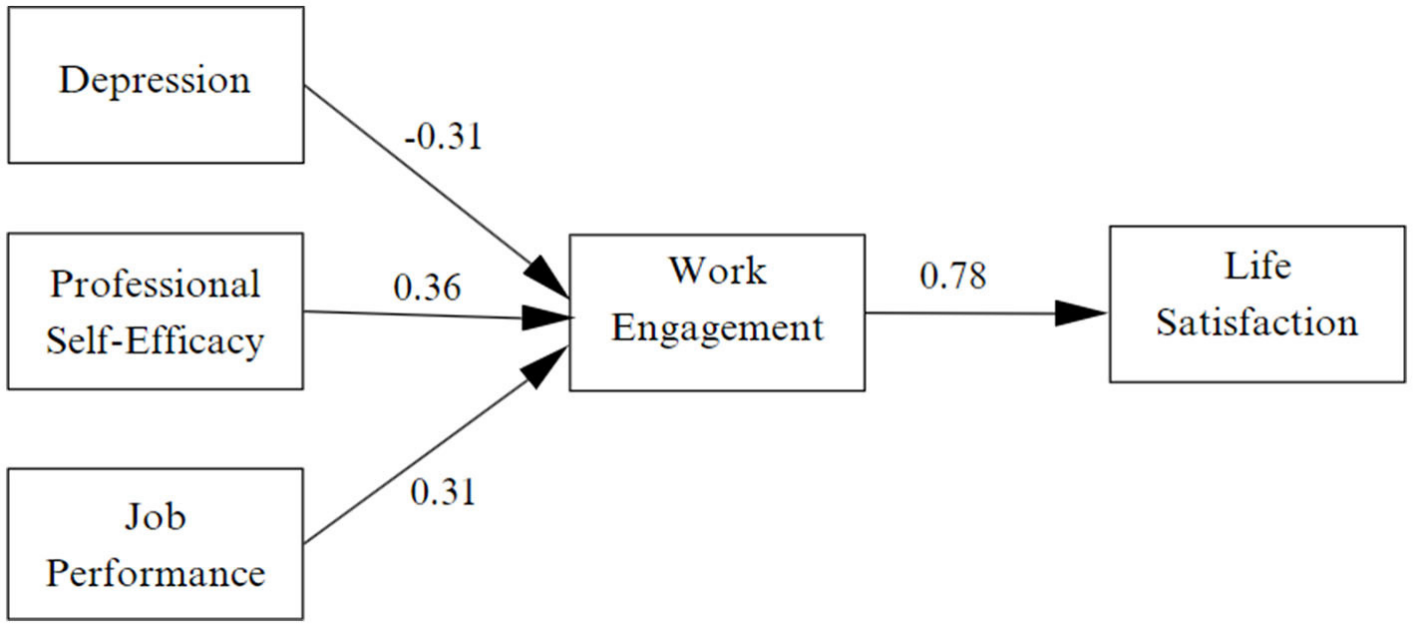
Results of the explanatory structural model of life satisfaction.

In the analysis of the theoretical model (Figure 2) an adequate fit was obtained, χ^2^ = 2,407.300, df = 692, *p* = 0.000, CFI = 0.92, TLI = 0.91, RMSEA = 0.07 (90% CI 0.06–0.07), SRMR = 0.07. With this result, H1 was confirmed, indicating that there is a negative relationship between depression and job engagement (β = −0.31, *p* < 0.001). H2 was also confirmed, showing a positive relationship between professional self-efficacy and job engagement (β = 0.36, *p* < 0.001). Similarly, H3 was validated, indicating a positive relationship between job performance and job engagement (β = 0.31, *p* < 0.001). Finally, H4 was confirmed, which shows a positive relationship between job engagement and life satisfaction (β = 0.78, *p* < 0.001).

### 3.3 Mediation model

For the mediation analysis, bootstrapping of 5,000 iterations was used, and these results are shown in Table 3. The mediating role of job engagement is confirmed in the relationship between depression (H5a; β = −0.12, *p* = 0.05), professional self-efficacy (H5b; β = −0.18, *p* < 0.001), job performance (H5c; β = −0.12, *p* < 0.001), and life satisfaction.

**Table 3:** Research hypotheses on indirect, total effects and their estimates.

**Path in the model**	**β**	***p*-value**	**95%CI**	
			**LL**	**UL**
**Indirect**				
H5a: Depression → job engagement → life satisfaction	−0.12	0.003	−0.226	−0.021
H5b: Professional self-efficacy → work engagement → life satisfaction	0.184	< 0.001	0.112	0.276
H5c: Work Performance → work engagement → life satisfaction	0.508	< 0.001	0.335	0.692
**Direct**				
Depression → work engagement	−0.22	0.02	−0.408	−0.037
Professional self-efficacy → work engagement	0.337	< 0.001	0.215	0.488
Work performance → work engagement	0.928	< 0.001	0.617	1.284
Work engagement → life satisfaction	0.547	< 0.001	0.432	0.657
**Total**				
Total effect of independent variables on life satisfaction	0.572	< 0.001	0.364	0.795

For the mediation analysis, bootstrapping with 5000 iterations was used, and these results show the indirect pathways of the structural model in Table 3. The mediating role of work engagement between the relationship of depression and life satisfaction (H5a; Depression → Work Engagement → Life Satisfaction) reveals a significant negative effect (β = −0.120, *p* = 0.023). Similarly, work engagement mediates the relationship between professional self-efficacy and life satisfaction (H5b; Professional Self-Efficacy → Work Engagement → Life Satisfaction) showing a significant positive effect (β = 0.184, *p* < 0.001). Furthermore, work engagement also mediates the relationship between work performance and life satisfaction (H5c: Work Performance → Work Engagement → Life Satisfaction), which is positive and significant (β = 0.508, *p* < 0.001). Regarding the direct effects, the pathway indicates that Depression has a direct and negative impact on Work Engagement (β = −0.220, *p* = 0.020), while it is confirmed that Professional Self-Efficacy improves Work Engagement (β = 0.337, *p* < 0.001). Additionally, a strong positive relationship is highlighted between Work Performance and Work Engagement (β = 0.928, *p* < 0.001), and finally, the pathway shows that higher Work Engagement leads to greater Life Satisfaction (β = 0.547, *p* < 0.001). The total effect (Total = 0.572, *p* < 0.001) synthesizes the combined impact of Depression, Professional Self-Efficacy, and Work Performance on Life Satisfaction, demonstrating how these variables, through their relationship with Work Engagement, integrally shape the experience of life satisfaction.

On the other hand, the R2 value for the variable Work Engagement is 0.704, indicating that the variables Depression, Professional Self-Efficacy, and Work Performance, together, explain 70.4% of the variation in Work Engagement. This high percentage of explained variance suggests that the model has considerable capacity to explain Work Engagement. As for the outcome variable Life Satisfaction, the R2 value is 0.516, indicating that Work Engagement explains 51.6% of the variation in Life Satisfaction. This is a significant percentage, indicating that the model is quite effective in capturing the factors that contribute to Life Satisfaction.

## 4 Discussion

Our findings add to the existing literature on the Job Demands-Resources (JD-R) theory by highlighting the mediating role of job engagement in the relationship between depression, professional self-efficacy, job performance, and life satisfaction in nurses. Analyzing these aspects within the intricate web of work and personal relationships provides an insight into how job demands, job and personal resources relate to job engagement and influence life satisfaction in nurses. This perspective not only allows us to better understand the reality of this population, but also to suggest and evaluate potential interventions to improve nurses' wellbeing and engagement with their work.

The results showed a negative relationship between depression and job engagement, which is consistent with previous research (5, 82). Nurses working in clinics and hospitals, grappling with high job demands such as heavy workloads, long working hours, lack of institutional support, and the pressures inherent in an extreme work environment, tend to experience higher levels of stress and emotional exhaustion (83). This increase in stress and emotional exhaustion can, unfortunately, lead to a higher risk of developing depression. Added to this is the constant exposure to stressful situations and the scarcity of resources which appear to exert a negative impact on nurses' ability to maintain positive job engagement (5). Thus, it is common to find that a high number of these health professionals experience depressive symptoms, a situation that is exacerbated by job uncertainty and the daily pressures they face in their work environment. In the context of the Job Demands-Resources (JD-R) theory, demands such as anxiety can be seen as significant predictors of job engagement. This study drew on this theory to better understand how job demands and resources can influence depression and job engagement in nurses (84).

The results also indicated a positive influence between professional self-efficacy and job engagement, which aligns with previous research that has shown a positive relationship between professional self-efficacy and job engagement (85, 86). When employees have a high degree of confidence in their abilities and competencies relevant to their assigned tasks, they engage more fully in their work, resulting in greater efficacy and job engagement (87). This self-efficacy plays a catalytic role, spurring employees to work with a dedication and persistence that enable them to overcome work problems and obstacles to successfully fulfill their tasks. This impetus translates into a job engagement that often goes beyond what is expected of them, generating organizational citizenship behaviors. This is where the power of engagement lies: not only does it enable employees to fulfill their work obligations, but it motivates them to exceed expectations (88). Similarly, our findings reaffirm that personal resources, such as self-efficacy, play a vital role in fostering job engagement. Employees who can effectively utilize these resources, such as control over work and involvement in decision-making, are more motivated and committed to the organization. In this respect, personal resources serve a dual role: on the one hand, they have an intrinsically motivating function, as they enhance individual learning and development (89). On the other hand, they extrinsically facilitate the achievement of work goals, becoming an indispensable tool for reaching professional targets. Thus, it is confirmed that nurses with a stronger sense of professional self-efficacy can face work challenges with more confidence and skill, which, in turn, can increase their job engagement (61, 84).

Also, the results indicated a positive influence between job performance and work engagement, suggesting that job performance is positively related to work engagement (54, 60). This positive influence can be attributed to the reality experienced by nurses: when they are immersed in their work with tenacity and passion to achieve their goals, their organizational commitment intensifies. This increase in commitment signifies a deeper immersion into the organizational structure and a sharper focus on their work, not only to grow personally but also to add value to the organization (54). Our analysis also builds on the job resources theory and the job demands-resources model, which suggest that work engagement improves job performance (90). In this context, the presence of employee commitment drives job performance. When nurses are committed to their work, this commitment translates into greater motivation, stronger dedication, and enthusiasm, which in turn translates into optimized job performance (60).

Our results decisively support the presence of a positive relationship between work engagement and life satisfaction, a finding that aligns with what was previously demonstrated (91, 92). This would be explained by the fact that those who experience positive feelings are more likely to experience job satisfaction. However, our study goes further, providing a deeper insight into how these factors intertwine.

Also, the results indicated a positive influence between work engagement and life satisfaction, which is consistent with previous research (91, 92). This is because people who experience more pleasant feelings tend to be more satisfied with their work. In addition, happy people tend to evaluate their skills and abilities positively, remember positive events more frequently, and share positive energy with their environment, which improves labor relations and satisfaction with work, colleagues, and the work environment in general (93). Thus, the work environment, including working conditions, organizational support, and leadership from supervisors, had a significant and positive effect on the nurses' affective commitment to the organization. A favorable work environment and adequate support from the organization and leaders can increase the emotional commitment of nurses to their work and the organization, which can have positive implications for their life satisfaction (92).

On the other hand, our results reveal the mediation of job engagement in the relationship between depression and life satisfaction, suggesting a complex intertwining of mental health, job commitment, and life happiness in the nursing profession. Following the framework of the Job Demands-Resources (JD-R) model, we have evaluated how the relationship between job engagement and life satisfaction is modulated by specific job resources and demands (90, 94). This model postulates that job resources can act as buffers against the harmful effects of job demands, such as stress and the cognitive and emotional demands of work. Thus, when job demands, both cognitive and emotional, increase, a negative relationship with job satisfaction is observed (94). In this sense, job engagement can mitigate this negative impact, acting as an essential mediator in this relationship. At the same time, nurses who have sufficient job resources, such as good teamwork, supervisor support, job control, and a sense of purpose in their work, experience less job pressure and, consequently, greater life satisfaction (95). Hence, our results also evidenced the mediation of job engagement in the relationship between job performance and life satisfaction. Likewise, the mediation of job engagement in the relationship between professional self-efficacy and life satisfaction was indicated. In line with this, personal resources such as self-efficacy, optimism, and determination to continue working, are factors that protect nurses from the negative impact of stress and contribute to greater job engagement and life satisfaction (96). Moreover, high job demands, such as psychosocial and emotional demands (depression), are linked with negative health outcomes, such as burnout and poorer self-rated health. Conversely, job resources, such as social support, cohesion, and rewards, are associated with positive health outcomes and a lower incidence of burnout (97). Nurses with high self-efficacy and positive personality traits, coupled with adequate access to job resources, are more likely to experience higher job engagement. This high job engagement, in turn, is associated with better job performance and greater life satisfaction (98). It's important to highlight that not only do job demands and job resources separately have favorable and unfavorable effects on job engagement, but the appropriate combination of job resources can influence the level of job engagement in stressful situations (64). By understanding this mechanism, interventions and policies can be developed to reduce turnover intentions and improve job satisfaction and emotional wellbeing of nurses (99).

### 4.1 Implications

Firstly, the importance of work engagement as a mediator between depression, self-efficacy, job performance, and life satisfaction is highlighted. This suggests that workplace interventions should aim to increase job engagement, which can act as a barrier against depression and can enhance performance and overall life satisfaction in nurses. For nursing professionals, the findings suggest that workplace training programs and interventions should focus on fostering self-efficacy and increasing nurses' ability to handle workload and stress. This might involve the implementation of resilience and stress management training programs, as well as strengthening social support networks in the workplace. Furthermore, health managers should focus on providing a work environment that promotes job engagement, with measures including appropriate task distribution, improved working conditions, and fostering a positive and supportive work atmosphere. Additionally, these findings underscore the need for policies to improve working conditions and reduce workload in the nursing sector. This could involve promoting flexible work policies, reducing the number of working hours, and enhancing mental health support for professionals. It's also essential to foster a work environment that promotes self-efficacy and provides nurses with the necessary skills and resources to cope with job demands. Finally, from a theoretical perspective, these findings enrich our understanding of the Job Demands-Resources (JD-R) theory by demonstrating how depression, self-efficacy, and job performance interact to influence life satisfaction through job engagement. Our results underline the importance of considering both positive aspects (like self-efficacy and job performance) and negative aspects (like depression) of job demands and resources, and how these can influence the health and wellbeing of nursing professionals.

Furthermore, we suggest additional research to explore how other factors, like social support and individual characteristics, might influence the relationship between depression, self-efficacy, job performance, job engagement, and life satisfaction. It would also be valuable to investigate how specific interventions based on the JD-R theory can enhance job engagement and life satisfaction in nurses. For example, interventions that increase job resources or decrease job demands could be explored, and how these interventions might improve engagement and job satisfaction. Lastly, future research could explore these themes in other professional contexts and in different cultures to better understand how the Job Demands-Resources (JD-R) theory applies in different contexts and can be used to improve the job health and wellbeing of employees in a variety of settings and cultures.

### 4.2 Limitations

Our findings emphasize the crucial mediating role of work engagement in linking depression, professional self-efficacy, job performance, and life satisfaction among nurses, shedding light on the complexities that nursing professionals face. These findings are particularly relevant given the essential role that nursing professionals play in our healthcare systems, a role that has become even more critical in light of recent challenges such as the global pandemic. Furthermore, the need for comprehensive strategies that address not only job demands but also ways to strengthen nurses' work and personal resources is underscored, aiming to improve their engagement, performance, and ultimately, their life satisfaction.

We acknowledge that our research is not without limitations. The first limitation pertains to the cross-sectional nature of our study. Future research could adopt a longitudinal approach to observe how these variables change over time and how interventions based on the JD-R theory might have a long-term impact, as suggested by Tims et al. (100). The second limitation is the sample selection. Although our participants represent a broad range of nurses from various specialties and work settings, our study was primarily focused on a specific geographical region. Future research could expand the sample to include nurses from different regions and cultural backgrounds to gain a more comprehensive picture of these relationships. Lastly, while we controlled for various confounding variables, there are other potential unobserved variables that might have influenced our results, such as individuals' personalities, their social support, or their personal mental health history. Future studies could include these and other relevant variables to provide a more robust analysis and to assess the possibility that Work Engagement acts as an effect modifier in the relationship between Depression and Life Satisfaction.

## 5 Conclusion

Our findings emphasize the crucial mediating role of job engagement in the connection between depression, professional self-efficacy, job performance, and life satisfaction in nurses, shedding light on the complexities that nursing professionals face. These findings are particularly pertinent, given the essential role nursing professionals play in our healthcare systems, a role that has become even more critical in light of recent challenges, such as the global pandemic. Furthermore, the need for comprehensive strategies that address not only job demands but also ways to strengthen the job and personal resources of nurses is underscored, with the aim of enhancing their engagement, performance, and ultimately, their life satisfaction.

## Data availability statement

The raw data supporting the conclusions of this article will be made available by the authors, without undue reservation.

## Ethics statement

The study was approved by the Ethics Committee of the Universidad Peruana Unión with code 2023-CEUPeU-033. The studies were conducted in accordance with the local legislation and institutional requirements. The participants provided their written informed consent to participate in this study.

## Author contributions

WM-G: Conceptualization, Formal analysis, Investigation, Software, Writing—original draft, Writing—review & editing. MV: Conceptualization, Data curation, Supervision, Validation, Visualization, Writing—original draft, Writing—review & editing. LS-S: Conceptualization, Resources, Software, Supervision, Validation, Writing—original draft. SM-G: Funding acquisition, Investigation, Resources, Software, Writing—review & editing. OR-L: Conceptualization, Resources, Software, Visualization, Writing—original draft. MM-G: Formal analysis, Investigation, Methodology, Validation, Visualization, Writing—review & editing.
